# Supplementary material for: Implications of Microstructure in Helium-Implanted Nanocrystalline Metals
Source: Materials (Basel). 2022 Jun 9;15(12):4092. doi: 10.3390/ma15124092 (PMC9231260; doi:10.3390/ma15124092)
Supplement: Supplementary file 1 [file materials-15-04092-s001.zip › materials-1730233-supplementary.pdf]

Supplementary Materials

Rate theory model of He partition in nanocrystalline metals

1 Overall kinetic model

The evolution equations for vacancies "V", self-interstitials "SIA", and He atoms "He" are shown in Table 2. These equations are coupled to one another via the reaction coefficients K (for vacancies and self-interstitials) and via the sink strength coefficients S_α (with $\alpha = \mathrm{V}, SIA, \mathrm{H}e$). Another important feature of the model is the existence of bubbles (i.e. Helium–vacancy complexes) that can absorb vacancies, self-interstitials, and He atoms. These are explained below.

Table S1: Differential equations for the irradiation species considered in the model.

**left-hand side**	**right-hand side**		
**Species**	**Source**	**Sinks**	**Reactions**
$\frac{dC_{\mathrm{H}e}}{dt}$	$G_{\mathrm{H}e}$	$-S_{\mathrm{He}}D_{\mathrm{He}}C_{\mathrm{He}}$	$-K_{\mathrm{V}-He}C_{\mathrm{V}}C_{\mathrm{He}}$
$\frac{dC_{\mathrm{V}}}{dt}$	G_{V}	$-S_{\mathrm{V}}D_{\mathrm{V}}C_{\mathrm{V}}$	$-K_{\mathrm{V}-SIA}C_{\mathrm{V}}C_{\mathrm{S}IA}$<br>$-K_{\mathrm{V}-He}C_{\mathrm{V}}C_{\mathrm{H}e}$
$\frac{dC_{SIA}}{dt}$	G_{SIA}	$-S_{SIA}D_{SIA}C_{SIA}$	$-K_{\mathrm{V}-SIA}C_{\mathrm{V}}C_{SIA}$
$\frac{dC_b}{dt}$	–	–	$K_{\mathrm{V}-He}C_{\mathrm{V}}C_{\mathrm{He}}$

The differential equation that controls bubble growth is written below:

$$\frac{dR}{dt} = \frac{\Omega_a}{R}\left(D_{\mathrm{V}}(C_{\mathrm{V}} - C_{\mathrm{V}}^0) - D_{SIA}C_{SIA}\right) \tag{1}$$

which reflects that bubble growth depends on the imbalance between the arrival fluxes of vacancies and interstitials. For this, it has been assumed that the defect flux equation $J_\alpha = -D_\alpha \frac{dC_\alpha}{dx}$ can be approximated as $J_\alpha \approx -\frac{D_\alpha C_\alpha}{R}$. Additionally, we consider the thermodynamic SIA concentration in equilibrium and near the void to be negligible. C_{V}^0 is the equilibrium vacancy concentration at the bubble–crystal interface.

$$C_{\mathrm{V}}^0 = C_{\mathrm{V}}^\infty \exp\left(-\frac{\Omega_a}{kT}\left(p_b - \frac{2\gamma}{R}\right)\right)$$

where $C_{\mathrm{V}}^\infty = \exp\left(-\frac{E_{\mathrm{V}}^f}{kT}\right)$, p_b is the internal (He) pressure of the void, γ is the bubble surface tension, and E_{V}^f is the vacancy formation energy. When the bubbles are in equilibrium, $C_{\mathrm{V}}^\infty \approx$

C_V^0, which is the assumption employed here.

2 Physical coefficients

The key parameters of the above equations can be separated into source terms, sink strengths, diffusion coefficients, and reaction coefficients.

2.1 Source terms

The parameters G_α represent the insertion fluxes of species α in the system. They are determined according to the irradiation conditions, and for ion irradiations, they are typically obtained with TRIM. The rates of primary damage production in cascades are given by:

$$G_V = G_{NRT}(1-\varepsilon_r)(1-\varepsilon_V) \tag{2}$$

$$G_{SIA} = G_{NRT}(1-\varepsilon_r)(1-\varepsilon_{SIA}) \tag{3}$$

where ε_r is the fraction of defects recombined in cascades relative to the NRT standard value, G_{NRT}, and ε_V and ε_{SIA} are the fractions of clustered vacancies and SIAs, respectively. The insertion rate of He atoms, G_{He}, is given simply by the ion current per unit volume. All the G_α are considered constants here.

From TRIM, the results were N_d =0.46 and 0.43 Frenkel pairs per ion per nm in Ni and Fe, respectively, at peak depth. To convert to damage rate:

$$G_{NRT} = N_d \phi_{He} \ [\mathrm{m}^{-3}\ \mathrm{s}^{-1}]$$

where ϕ_{He} is the He-ion irradiation flux[1]. For He-ion fluences of 2.8×10^{21} and 5.0×10^{21} m^{-2} over 3200 s of irradiation, one gets fluxes of $\approx 8.8 \times 10^{17}$ and 1.6×10^{18} $\mathrm{m}^{-2}\ \mathrm{s}^{-1}$ for Fe and Ni, respectively. The concentration of He ions at a depth of 40 nm is approximately 1.2×10^5 (atoms/cm^3)/(atoms/cm^2). Multiplying by the respective fluxes gives the corresponding G_{He} for Fe and Ni:

$$G_{He} = 1.2 \times 10^5 \cdot 8.8 \times 10^{13} = 1.06 \times 10^{19} \ [\mathrm{cm}^{-3}\ \mathrm{s}^{-1}] \text{ (in Fe)}$$

$$G_{He} = 1.2 \times 10^5 \cdot 1.6 \times 10^{14} = 1.92 \times 10^{19} \ [\mathrm{cm}^{-3}\ \mathrm{s}^{-1}] \text{ (in Ni)}$$

2.2 Sink strengths

The system is assumed to be a nanocrystal with grain size d and with no ingrown dislocations. The only sinks for defects are thus grain boundaries and bubbles forming in the interior of the grains. The general expression for the sink strength of a species α is:

$$S_\alpha = z_\alpha(S_b + S_{GB}) = z_\alpha\left(4\pi R C_b + \frac{6\sqrt{4\pi R C_b}}{d}\right)$$

[1] When converting to dpa/s, divide this quantity by ρ, the atomic density of Ni or Fe

where z_α is a bias factor. Note that this expression appears in all the kinetic equations in Table 2 and is connected to Equation (1) via the radius R.

2.3 Reaction constants

The reaction constants K follow a standard Smoluchowski's form for all species:

$$K_{\alpha\beta} = 4\pi(r_\alpha + r_\beta)(D_\alpha + D_\beta)$$

where r_α and D_α are the size and diffusivity of species α, respectively. In our case, only $K_{\mathrm{V}-SIA}$ and $K_{\mathrm{V}-He}$ are relevant. Noting that $D_{SIA} \gg D_{\mathrm{V}}$ and that $r_{SIA} \approx r_{\mathrm{V}} = 2r_a$ ($r_a = (3\Omega_a/4\pi)^{1/3}$), we can write:

$$K_{\mathrm{V}-SIA} \approx 16\pi r_a D_{SIA}$$

and, similarly:

$$K_{\mathrm{V}-He} \approx 16\pi r_a D_{\mathrm{He}}$$

2.4 Diffusivities

Finally,

$$D_\alpha = D_0^\alpha \exp\left(-\frac{E_m^\alpha}{kT}\right)$$

where D_0^α and E_m^α are the prefactor and migration energy of species α.

3 Model parameters

Table S2: Parameters needed in the model described above.

Parameter	Fe	Ni
D_0^{V} [m^2 s^{-1}]	7.90×10^{-7}	1.33×10^{-4} [2]
D_0^{SIA} [m^2 s^{-1}]	1.30×10^{-8}	1.05×10^{-7} [3]
D_0^{He} [m^2 s^{-1}]	2.80×10^{-8}	5.22×10^{-7}
E_m^{V} [eV]	0.60	1.3 [4]
E_m^{SIA} [eV]	0.25	0.11 [5]

[2] https://onlinelibrary.wiley.com/doi/epdf/10.1002/pssb.2220780230, https://doi.org/10.1016/j.msea.2014.08.054.

[3] https://iopscience.iop.org/article/10.1088/2053-1591/aab418/meta

[4] https://onlinelibrary.wiley.com/doi/abs/10.1002/1521-3951(200211)234:2%3C506::AID-PSSB50%3E3.0.CO;2-Q

[5] https://arxiv.org/pdf/1607.04667.pdf

E_m^{He} [eV]	0.06	0.44 [6] (0.14 [7])
E_f^{V} [eV]	1.7	1.5 [8]
E_b^{GB} [eV]	0.8	1.0
d [nm]	100	40
Ω_a [m $^{-3}$]	1.2×10^{-29}	1.1×10^{-29} [9]
z_{V}	1.0	1.0
z_{SIA}	1.1	1.1
z_{He}	1.1	1.1
ε_{r}	0.35[10]	0.4
ε_{SIA}	0.5[11]	0.5[12]
ε_{V}	0.3[13]	0.2
N_d [m $^{-1}$]	4.8×10^{8}	4.6×10^{8}
ρ [m $^{-3}$]	8.5×10^{28}	9.1×10^{28}
T [K]	873	1073
ϕ_{He} [s $^{-1}$ m $^{-2}$]	8.75×10^{17}	1.56×10^{18}
G_{He} [s $^{-1}$ m $^{-3}$]	1.06×10^{25}	1.92×10^{25}
k [eV·K $^{-1}$]	8.615×10^{-5}	

4 Finite difference solution procedure

We solve the above system of equations using a discrete finite difference scheme. The sequence of equations is:

$$R^{n+1} = R^n + \frac{\delta t \Omega_a}{R^n}\left(D_{V}\left(C_{V}^{n} - C_{V}^{eq}\right) - D_{SIA}C_{SIA}^{n}\right) \tag{4}$$

$$C_b^{n+1} = C_b^n + \delta t K_{V-He} C_{V}^{n} C_{He}^{n} \tag{5}$$

$$S_\alpha^{n+1} = z_\alpha 4\pi R^{n+1} C_b^{n+1} + \frac{6 z_\alpha \sqrt{4\pi R^{n+1} C_b^{n+1}}}{d} \tag{6}$$

[6] https://www.sciencedirect.com/science/article/pii/S0927025615003304

[7] https://www.sciencedirect.com/science/article/pii/0022311583900764

[8] https://arxiv.org/pdf/1607.04667.pdf

[9] https://www.ncbi.nlm.nih.gov/pmc/articles/PMC6153149/

[10] https://www.sciencedirect.com/science/article/pii/S0022311503003593

[11] http://li.mit.edu/Stuff/CNSE/Paper/Bacon00GaoJNM.pdf

[12] https://link.springer.com/article/10.1134/S0031918X20010196

[13] https://www.sciencedirect.com/science/article/pii/S0022311503003593

$$C_{\mathrm{V}}^{n+1} = C_{\mathrm{V}}^{n} + \delta t[G_{\mathrm{V}} - S_{\mathrm{V}}^{n+1} D_{\mathrm{V}} C_{\mathrm{V}}^{n} - (K_{\mathrm{V}-SIA} C_{\mathrm{SIA}}^{n} + K_{\mathrm{V}-He} C_{\mathrm{He}}^{n}) C_{\mathrm{V}}^{n}] \tag{7}$$

$$C_{\mathrm{SIA}}^{n+1} = C_{SIA}^{n}[1 - \delta t(S_{\mathrm{SIA}}^{n+1} D_{SIA} + K_{\mathrm{V}-SIA} C_{\mathrm{V}}^{n})] + G_{SIA}\delta t \tag{8}$$

$$C_{He}^{n+1} = C_{\mathrm{He}}^{n} + \delta t[G_{\mathrm{He}} - S_{\mathrm{He}}^{n+1} D_{\mathrm{He}} C_{\mathrm{He}}^{n} - K_{\mathrm{V}-He} C_{\mathrm{V}}^{n} C_{\mathrm{He}}^{n}] \tag{9}$$

This updates the concentrations of all species in time step $n+1$ from quantities from time step n. The initial conditions are simply:

$$C_b^0 = C_{\mathrm{V}}^0 = C_{\mathrm{SIA}}^0 = C_{\mathrm{He}}^0 = 0$$

i.e. at $t = 0$, all species concentrations are zero. To avoid the singularity in Equation (1), one can start with $R^0 = r_a$.

5 Adding thermal dissociation

For this, a new "species" must be created, representing He atoms at the grain boundaries sinks). The accumulation there is governed by the equation:

$$\frac{dC_{\mathrm{He}}^{GB}}{dt} = \left(\frac{6\sqrt{4\pi R C_b}}{d}\right) D_{\mathrm{He}} C_{\mathrm{He}} - \left(\frac{\pi d^2}{r_a^4}\right) D_{\mathrm{He}} C_{\mathrm{He}}^{GB} \exp\left(-\frac{E_b^{\mathrm{GB}}}{kT}\right) \tag{10}$$

He from the grain boundary can then be thermally desorbed and put back in solution in the bulk. With this, the revised equation for C_{He} is:

$$\frac{dC_{\mathrm{He}}}{dt} = \left(G_{\mathrm{He}} + \left(\frac{\pi d^2}{r_a^4}\right) D_{\mathrm{He}} C_{\mathrm{He}}^{GB} \exp\left(-\frac{E_b^{\mathrm{GB}}}{kT}\right)\right) - S_{\mathrm{He}} D_{\mathrm{He}} C_{\mathrm{He}} - K_{\mathrm{V}-He} C_{\mathrm{V}} C_{\mathrm{He}} \tag{11}$$

where the second term in parentheses on the r.h.s. of the equation is the new "source" term defined by the binding energy E_b^{GB}. In finite differences, Equation (11) is expressed as:

$$\begin{aligned} C_{\mathrm{He}}^{n+1} &= C_{\mathrm{He}}^{n}[1 - \delta t(S_{\mathrm{He}}^{n+1} D_{\mathrm{He}} C_{\mathrm{He}}^{n} + K_{\mathrm{V}-He} C_{\mathrm{V}}^{n} C_{\mathrm{He}}^{n})] + \\ &+ \delta t \left[G_{\mathrm{He}} + \frac{\pi d^2 D_{\mathrm{He}} \left(C_{\mathrm{He}}^{GB}\right)^{n+1}}{r_a^4} \exp\left(-\frac{E_b^{\mathrm{GB}}}{kT}\right)\right] \end{aligned} \tag{12}$$

which replaces Equation (9) above and where:

$$\left(C_{\mathrm{He}}^{\mathrm{GB}}\right)^{n+1} = \left(C_{\mathrm{He}}^{\mathrm{GB}}\right)^{n} + \delta t D_{\mathrm{He}} \left[\left(\frac{6\sqrt{4\pi R^n C_b^n}}{d}\right) C_{\mathrm{He}}^{n} - \left(\frac{\pi d^2}{r_a^4}\right) \exp\left(-\frac{E_b^{\mathrm{GB}}}{kT}\right) \left(C_{\mathrm{He}}^{GB}\right)^{n}\right]$$

must be inserted between eqs. (6) and (9) with $\left(C_{\mathrm{He}}^{GB}\right)^0 = 0$. Dissociation from bubbles is formulated in similar way (omitted here for clarity).
